# Acquisition of yersinia murine toxin enabled *Yersinia pestis* to expand the range of mammalian hosts that sustain flea-borne plague

**DOI:** 10.1371/journal.ppat.1009995

**Published:** 2021-10-14

**Authors:** David M. Bland, Adélaïde Miarinjara, Christopher F. Bosio, Jeanette Calarco, B. Joseph Hinnebusch

**Affiliations:** Laboratory of Bacteriology, Rocky Mountain Laboratories, National Institute of Allergy and Infectious Diseases, NIH, Hamilton, Montana, United State of America; University of Missouri, UNITED STATES

## Abstract

Yersinia murine toxin (Ymt) is a phospholipase D encoded on a plasmid acquired by *Yersinia pestis* after its recent divergence from a *Yersinia pseudotuberculosis* progenitor. Despite its name, Ymt is not required for virulence but acts to enhance bacterial survival in the flea digestive tract. Certain *Y*. *pestis* strains circulating in the Bronze Age lacked Ymt, suggesting that they were not transmitted by fleas. However, we show that the importance of Ymt varies with host blood source. In accordance with the original description, Ymt greatly enhanced *Y*. *pestis* survival in fleas infected with bacteremic mouse, human, or black rat blood. In contrast, Ymt was much less important when fleas were infected using brown rat blood. A *Y*. *pestis* Ymt^−^ mutant infected fleas nearly as well as the Ymt^+^ parent strain after feeding on bacteremic brown rat blood, and the mutant was transmitted efficiently by flea bite during the first weeks after infection. The protective function of Ymt correlated with red blood cell digestion kinetics in the flea gut. Thus, early *Y*. *pestis* strains that lacked Ymt could have been maintained in flea-brown rat transmission cycles, and perhaps in other hosts with similar blood characteristics. Acquisition of Ymt, however, served to greatly expand the range of hosts that could support flea-borne plague.

## Author summary

The bacterium *Yersinia pestis* causes highly lethal bubonic plague in a wide variety of mammals and is transmitted primarily by the bites of infected fleas. During its recent evolutionary divergence from *Yersinia pseudotuberculosis*, a mild pathogen incapable of flea-borne transmission, *Y*. *pestis* acquired a new gene that encodes a phospholipase enzyme called Yersinia murine toxin (Ymt). This was a critical step in the transition to an insect-borne life cycle as it was reported that Ymt activity greatly enhances bacterial survival in the flea gut. Recent genomic sequencing of ancient *Y*. *pestis* strains revealed that some lacked Ymt, leading to the conclusion that these strains were not transmitted by flea bite. Here, we report that the importance of Ymt for survival in the flea is greatly dependent on host blood source. Ymt is required if fleas take up *Y*. *pestis* in mouse, human, or black rat blood, but is not required if brown rat blood is used. We conclude that ancient *Y*. *pestis* strains lacking Ymt could have circulated in certain flea-rodent transmission cycles. Acquisition of Ymt, however, enabled *Y*. *pestis* to greatly expand its host range to an ecologically broad range of mammals and their fleas.

## Introduction

*Yersinia pestis* evolved from the closely related *Yersinia pseudotuberculosis*, a food-borne pathogen that generally causes self-limiting enteric disease, within the last 6,000 years [1,2]. Making only 5 specific genetic changes to *Y*. *pseudotuberculosis* results in a strain able to produce a transmissible infection in the flea [3]. One key gene acquired during transition to the flea-borne life cycle encodes Yersinia murine toxin (Ymt), a phospholipase D enzyme that has an important role in the ability of *Y*. *pestis* to colonize the flea midgut [4]. Ymt is encoded on the *Y*. *pestis*-specific pMT1 plasmid, which was acquired through horizontal gene transfer [5]. Ymt was once believed to be an important virulence factor in the mammalian host, as Ymt-enriched protein fractions are highly lethal to mice and rats [6,7]. However, Ymt is not required for typical plague disease progression and virulence and the LD50 of a Ymt-negative strain in mice is equivalent to that of wild-type *Y*. *pestis* [8]. Murine toxicity of Ymt is likely related to its ability to act as a β-andrenergic-blocking agonist in mice and rats [9,10], but toxicity is not observed in other mammals such as guinea pigs, rabbits, dogs, and primates [11]. Application of molecular Koch’s postulates to a standardized flea model of *Y*. *pestis* infection revealed that Ymt’s true biological function is to enhance bacterial survival in the flea midgut, significantly improving the ability of the plague bacillus to stably infect and be transmitted by its vector [4,12].

In the original characterization, a Ymt mutant was rapidly eliminated from ~90% of *Xenopsylla cheopis* fleas, and those few fleas with chronic infections had reduced bacterial burdens in which only the proventricular valve in the foregut (and not the midgut) was colonized. The incidence of transmission-enhancing proventricular blockage due to *Y*. *pestis* biofilm accumulation was correspondingly rare, indicating low potential for Ymt^−^ strains to be vectored by fleas [4]. The *Y*. *pestis* Ymt mutant was eliminated from fleas within the first 24h following uptake in a blood meal, preceded by conversion of the bacilli to an atypical spheroplast morphology in the midgut [4]. Bacterial spheroplast formation usually indicates damage to, or loss of, the bacterial outer membrane and a reduction in osmotolerance. Addition of recombinant Ymt protein to the infectious blood meal did not protect mutant bacilli from clearance, and in fleas coinfected with Ymt^−^ and Ymt^+^ *Y*. *pestis*, Ymt^−^ bacteria persisted in the midgut only if they were embedded within a biofilm of Ymt^+^ bacilli. Immunohistochemistry and immunoassays of culture supernatants indicate that Ymt is not secreted and is released only upon cell lysis [8]. Collectively, current data indicate that the Ymt phospholipase exerts its protective function intracellularly and that Ymt mutant bacteria are better able to survive in the flea gut if protected from the surrounding digestive and/or immunological milieu of the midgut [4].

In seeming contradiction to the rapid clearance phenotype observed for Ymt mutant bacteria [4], a separate study showed that Ymt^−^ *Y*. *pestis* could survive in and be transmitted by fleas up to 3 days after infection nearly as efficiently as the parental strain [13]. Notably, the study indicating that Ymt was dispensable for this early-phase transmission used brown rat (*Rattus norvegicus*) blood for the infectious blood meal [13], whereas the study demonstrating rapid clearance of the Ymt mutant from fleas used mouse blood [4]. Recently, we have shown that the source of infectious host blood alters the nature of the *Y*. *pestis* infection in the flea foregut [14]. Specifically, the slow digestion rate of brown rat blood and the relative insolubility of its hemoglobin promotes more rapid and extensive foregut infection (proventriculus and esophagus) in which partially digested blood meal contents mixed with *Y*. *pestis* are refluxed from the midgut into the esophagus; a phenomenon we have termed post-infection esophageal reflux (PIER) [14]. PIER-inducing blood sources reduce the time required for some rodent fleas to become infectious; increasing the number of bacilli transmitted during the first few days following an infectious blood meal. Because brown rat blood promotes infection of the esophagus and the bactericidal agent of Ymt^−^ strains is believed to be generated during blood digestion in the midgut, we thought Ymt^−^ *Y*. *pestis* might be better able to survive in fleas if PIER-inducing blood sources were used for the infection. To test this hypothesis and evaluate the permissiveness of different host blood sources to flea colonization, we infected rodent fleas with either wild-type or Ymt^−^ *Y*. *pestis* suspended in blood collected from mice, brown rats, black “roof” rats (*Rattus rattus*), or humans.

In resolution of the seemingly contradictory results, we found that Ymt mutant *Y*. *pestis* can chronically infect and be transmitted by the rodent fleas *X*. *cheopis* and *Oropsylla montana* at much higher levels if brown rat blood is used for the infectious blood meal than if mouse, human, or black rat blood is used. Our results suggest that ancestral *Y*. *pestis* strains lacking Ymt could have been maintained in flea-borne transmission cycles involving brown rats and perhaps other mammals with similarly permissive blood biochemistry. Acquisition of Ymt, however, fortified that ability and allowed *Y*. *pestis* to greatly expand its host range to involve many other mammals and their fleas, resulting in strong positive selective pressure for the Ymt^+^ lineage.

## Results

### The *Y*. *pestis* Ymt mutant induces PIER in fleas following an infectious brown rat blood meal

When fleas ingest *Y*. *pestis* suspended in blood that is digested relatively slowly and is characterized by a poorly soluble hemoglobin molecule, many of them exhibit post-infection esophageal reflux (PIER) [14]. The foregut of these fleas contains a mixture of partially digested blood components and *Y*. *pestis* aggregates that extends from the proventriculus forward into the esophagus within 24 h after an infectious blood meal. This phenomenon is seen following infections using brown rat and guinea pig blood, but not when mouse or gerbil blood is used [14]. Because digestive enzymes are likely not present at high concentration in the foregut, we hypothesized that bacteria aggregated there would be protected from bactericidal agents generated in the midgut, and that the foregut thus might provide a niche for Ymt^−^ *Y*. *pestis* to temporarily colonize if PIER-inducing blood is used for the infection.

**Fig 1:**
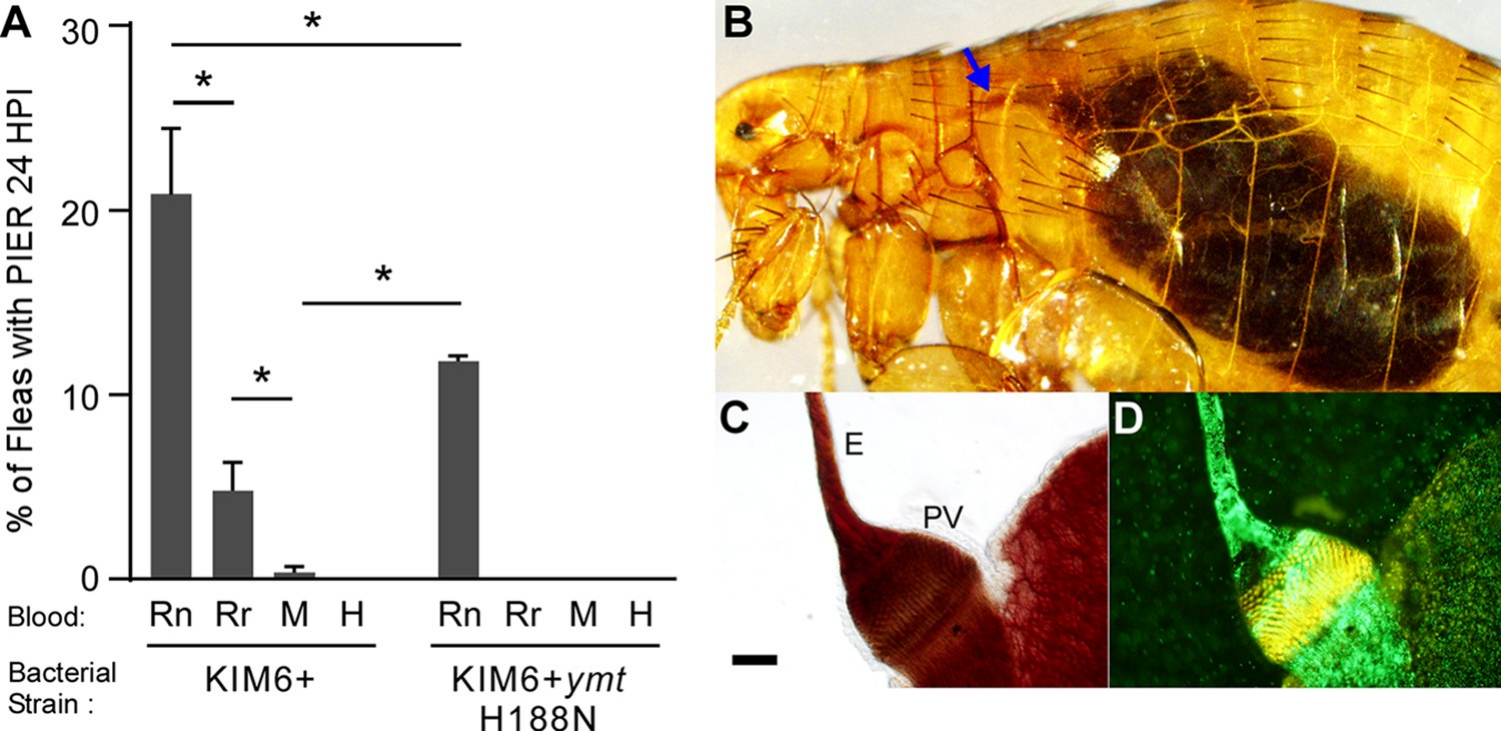
A *Y*. *pestis* Ymt mutant induces PIER in *X*. *cheopis* fleas when brown rat blood is used for the infectious blood meal. A) Incidence of post-infection esophageal reflux (PIER) in groups of 25 to 220 *X*. *cheopis* fleas 24 h after feeding on brown rat (Rn), black rat (Rr), mouse (M), or human blood (H) containing 1.5 x 10^8^–1.1 x 10^9^ CFU/ml KIM6+ or KIM6+*ymt*H188N *Y*. *pestis*. Bars show the mean and standard error of 3 independent experiments (n = 164–438 mixed sex fleas). *p < 0.005 by chi-square test. B) Female *X*. *cheopis* with PIER 24 h after feeding on black rat blood containing GFP-positive *Y*. *pestis* KIM6+; blue arrow indicates where blood and *Y*. *pestis* has been refluxed from the proventriculus and/or midgut into the esophagus. C) light and D) fluorescence microscopy images of the digestive tract dissected from this flea showing the presence of partially digested blood components and bacteria in the proventriculus (PV) and esophagus (E). Scale bar = 50 μm.

To determine if PIER induction occurs and could provide protection to Ymt^−^ strains, we infected *X*. *cheopis* fleas using one of four blood sources (brown rat, black rat, mouse, and human) and screened them 24 h later for PIER (Fig 1). Consistent with our previous study, PIER was evident in ~20% of fleas infected with wild-type *Y*. *pestis* KIM6+ using brown rat blood, but not when mouse blood was used. PIER was also induced in fleas infected using black rat blood, but at lower incidence (~5%) than for brown rat blood. Fleas infected using human blood did not develop PIER (Fig 1A). Notably, PIER was also observed in fleas following infection with Ymt^−^ *Y*. *pestis* in brown rat blood (but not black rat blood), but only about half as often as in fleas infected with the parental KIM6+ strain (Fig 1A).

As in our previous study, PIER correlated with the presence of hemoglobin crystals, partially digested red blood cell stroma, and *Y*. *pestis* in the proventriculus and esophagus of fleas (Fig 1B–1D) [14]. Hemoglobin crystals were commonly observed in the midgut of infected fleas when black rat blood was used for the infectious blood meal but appeared to be more soluble than brown rat hemoglobin crystals. Black rat hemoglobin crystals typically had a long rod-like shape and rapidly dissolved in the PBS we used to prepare wet mounts of infected flea digestive tracts, making them difficult to image and possibly causing us to underestimate their prevalence. In addition, unlike brown rat blood [14], hemolysis of black rat red blood cells in water did not result in hemoglobin crystallization. Hemoglobin crystals were not observed in the gut of fleas infected using mouse blood [14], and rarely observed in fleas infected using human blood.

Collectively, these data suggest that Ymt^−^ *Y*. *pestis* can colonize the flea foregut, induce PIER, and potentially be protected from elimination when brown rat blood is used for the infectious blood meal.

### Blood source affects colonization of rodent fleas by Ymt-deficient *Y*. *pestis*

**Fig 2:**
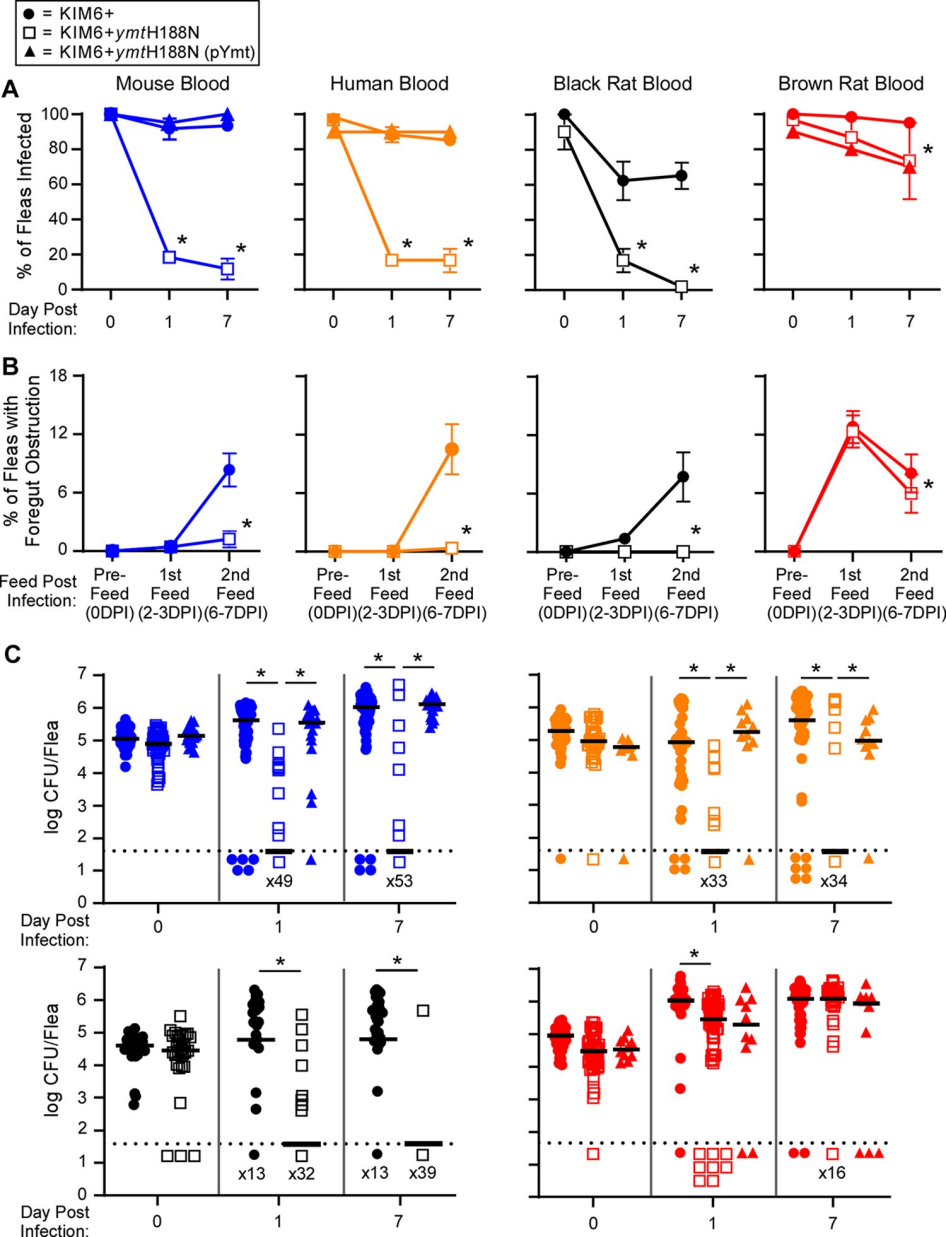
Ymt^−^ and Ymt^+^ *Y*. *pestis* colonize female *X*. *cheopis* similarly when brown rat blood is used for the infectious blood meal, but not if mouse, human, or black rat blood are used. Groups of female *X*. *cheopis* fleas that fed on mouse (blue), black rat (black), human (orange), or brown rat (red) blood containing 1.5x10^8^–1.1x10^9^ CFU/ml *Y*. *pestis* KIM6+, KIM6+*ymt*H188N, or KIM6+*ymt*H188N (pYmt) were scored for 1 week for A) the percentage of fleas that remained infected; B) the percentage that developed obstruction of the foregut (partial or complete blockage) that interfered with normal blood-feeding; and C) bacterial burden. Data are cumulative from 3 (KIM6+ and KIM6+*ymt*H188N groups) or 1 (KIM6+*ymt*H188N(pYmt) groups) independent experiments. Samples consisted of 7–20 female (A and C) or 25–220 fleas (roughly equal numbers of males and females; B) per experiment. The mean and standard error (A, B) or median (C) are indicated. *p < 0.05 by chi-square (A, B) or by Kruskal-Wallis test with Dunn’s post-test (mouse, human, and brown rat groups) or Mann-Whitney test (black rat group) (**C**). Dotted lines indicate the limit of detection (40 CFU). KIM6+*ymt*H188N(pYmt) was not used for black rat blood infections due to the limited availability of this blood.

To determine whether blood source and PIER affect the overall ability of the Ymt mutant to colonize the flea, *X*. *cheopis* were fed mouse, human, black rat, or brown rat blood containing ~ 5x10^8^ CFU/ml *Y*. *pestis* KIM6+, KIM6+*ymt*H188N, or KIM6+*ymt*H118N (pYmt). Infected fleas subsequently received two sterile maintenance blood meals over the course of 1 week to evaluate their potential to become blocked. Replicating previously published results [4], 80–90% of female fleas infected with the Ymt mutant in mouse blood cleared the infection within 24 h, whereas strains that produce the functional Ymt enzyme were rarely cleared by fleas during the first week (Fig 2A). The foregut of the few fleas that remained infected with the Ymt mutant in mouse blood rarely became obstructed by a bacterial mass (partially or fully blocked) during the first week of the infection and had reduced bacterial burdens (Fig 2B and 2C). Comparable results were observed for fleas infected with the Ymt mutant in human or black rat blood (Fig 2). In contrast, the average infection rate (73%) and median bacterial burden (1.2 x10^6^ CFU) after 1 week for fleas infected with the Ymt mutant in brown rat blood were only modestly lower than the infection rate (mean 95%) and bacterial burden (median 1.5 x10^6^ CFU) of fleas infected with the wild-type parent strain. Furthermore, fleas infected with the Ymt mutant in brown rat blood developed proventricular blockage at a rate similar to that of fleas infected with the parent strain at the first feeding following infection (12% vs 13%) and at a slightly reduced rate (6% vs 8%) after the second feeding (Fig 2B). To verify that these results were not unique to *X*. *cheopis* rat fleas, we replicated the experiments using mouse and brown rat blood with *Oropsylla montana*, a North American ground squirrel flea. The results mirrored those seen for *X*. *cheopis*: the Ymt mutant was rapidly cleared from *O*. *montana* fleas infected using mouse blood, but those infected using brown rat blood had infection rates, bacterial burdens, and proventricular obstruction rates that were equivalent to or only slightly reduced from wild-type levels (S1 Fig).

These results were surprising, because although a minority of the fleas infected using brown rat blood developed PIER (10–20%), much higher proportions (30–100%) remained infected for up to 1 week. Thus, it seems unlikely that PIER alone accounted for the high rates of flea colonization observed for the Ymt mutant-brown rat blood infections. However, the data provide insight into a previous report that the Ymt mutant can be as efficiently transmitted as its wild-type parent during the early phase when brown rat blood is used for the infectious blood meal [13]. In sum, our results show that the previously reported lability of Ymt^−^ *Y*. *pestis* in the flea gut varies depending on the infectious blood source. This mutant fares poorly after infectious mouse, black rat, or human blood meals, but survives much better after brown rat infectious blood meals. This effect is conserved in two rodent flea species from distinct taxonomic families.

### The protective role of Ymt is more pronounced in female fleas than in male fleas

**Fig 3:**
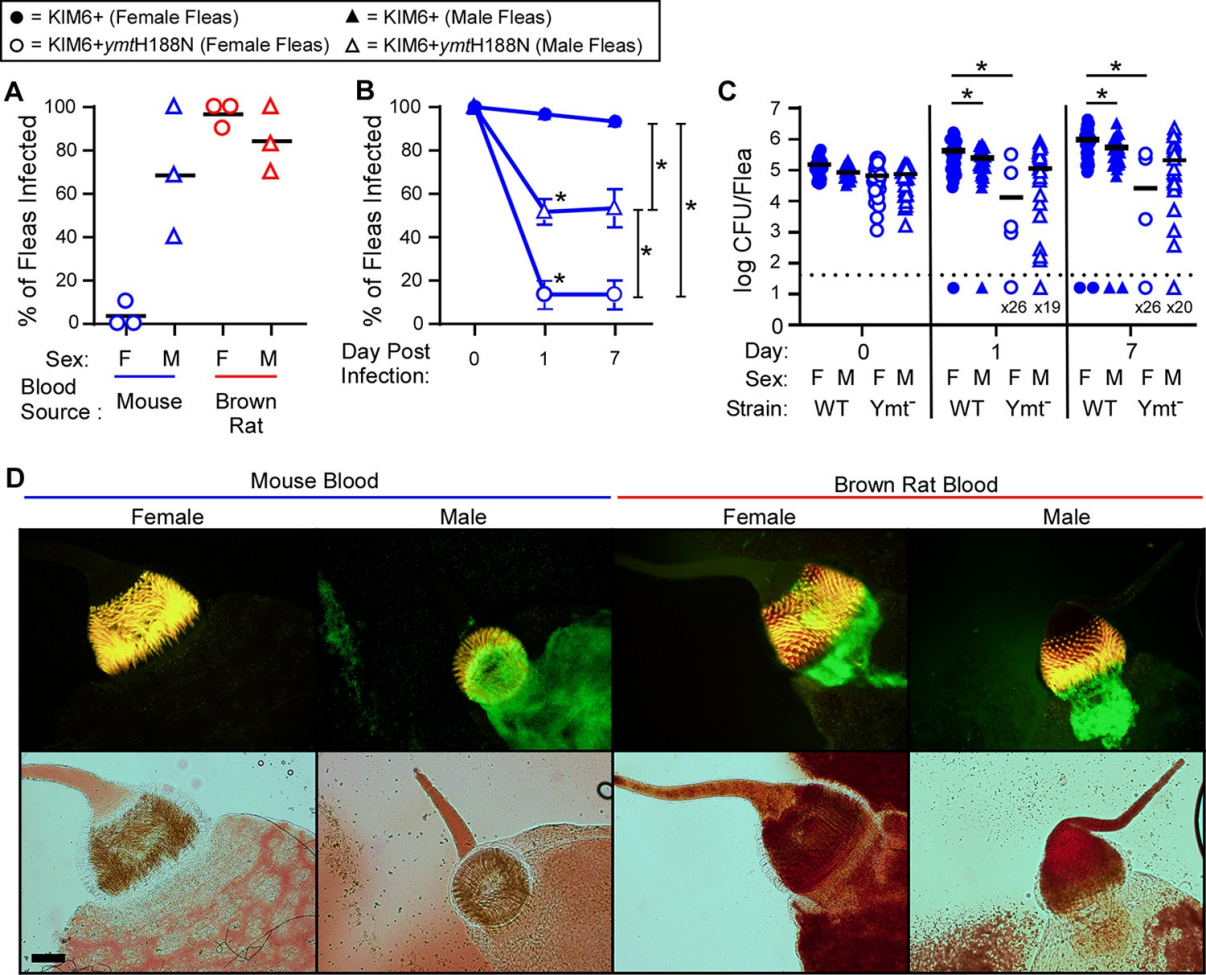
The *Y*. *pestis* Ymt mutant colonizes male fleas more efficiently than females following infection using mouse blood. Infection rates for groups of female or male *X*. *cheopis* infected using mouse blood (blue symbols) or brown rat blood (red symbols) containing 1x10^8^–5.7x10^8^ CFU/ml GFP-positive KIM6+ or KIM6+*ymt*H188N *Y*. *pestis* were determined 1 day after infection by fluorescence microscopy of dissected flea digestive tracts A); or 0, 1, and 7 days after infection by CFU counts from individual triturated fleas (B, C). For A, each symbol represents the percentage of fleas containing GFP+ bacteria in their digestive tract. n = 4–10 fleas of each sex in 3 independent experiments (Table 1). For B and C, the mean and standard error (B) or median (C) of pooled data from 3 independent experiments for groups of 5–20 fleas infected using mouse blood are shown. *p < 0.05 by chi-square test (B) or two-way ANOVA with Tukey’s post-test (C). D) Examples of the foregut infection in female or male *X*. *cheopis* 1 day after ingesting KIM6+*ymt*H188N *Y*. *pestis* suspended in mouse blood (Left) or brown rat blood (Right). Scale bar = 50 μm.

In the original characterization of the Ymt mutant strain in fleas, infection rates were determined only for female *X*. *cheopis* fleas infected using mouse blood [4], and the rates in Fig 2 were also based on female fleas. Because the metabolism and physiology of insects is not identical between sexes, we evaluated infection rates separately for male and female fleas infected with Ymt mutant *Y*. *pestis* using either mouse or brown rat blood. Unexpectedly, when mouse blood was used for the flea infection, 61% of male fleas remained infected after 24 h, whereas only a single female (4%) had evidence of GFP+ bacteria in the digestive tract (Fig 3A and Table 1). In contrast, when brown rat blood was used for the flea infections, male and female fleas had equivalently high rates of *Y*. *pestis* colonization and 25% (including examples of both sexes) had more severe bacterial infections in the proventriculus (Table 1). These data show an enhanced capacity for Ymt^−^ bacteria to survive in the male flea midgut.

**Table 1:** Flea Dissection Summary.

Blood Source /Experiment	% Fleas Infected (KIM6+*ymt*H188N)		Bacteria Present In:			PV Infection Severity		
*X*. *cheopis*	Male	Female	PV+ MG	PV only	MG only	Light	Moderate	Heavy
Mouse Blood #1	67% (9)	10% (10)	66%	0%	33%	67%	0%	0%
Mouse Blood #2	100% (4)	0% (5)	100%	0%	0%	100%	0%	0%
Mouse Blood #3	40% (10)	0% (9)	50%	0%	50%	50%	0%	0%
Total/Average	**61% (14/23)**	**4% (1/24)**	**67% (10/15)**	**0% (0/15)**	**33% (5/15)**	**67% (10/15)**	**0% (0/15)**	**0% (0/15)**
Rat Blood #1	70% (10)	100% (10)	82%	6%	12%	47%	35%	6%
Rat Blood #2	83% (6)	100% (6)	100%	0%	0%	73%	27%	0%
Rat Blood #3	100% (10)	90% (10)	100%	0%	0%	100%	11%	0%
Total/Average	**85% (22/26)**	**96% (25/26)**	**94% (44/47)**	**2% (1/47)**	**4% (2/47)**	**70% (33/47)**	**23% (11/47)**	**2% (1/47)**

PV = proventriculus, MG = midgut. Numbers in parentheses indicate flea sample sizes.

The infection status and bacterial load of fleas 1 and 7 days after infection was determined to assess whether the Ymt mutant persisted in male fleas infected using mouse blood. Significantly more males (40–70%) than females (0–25%; Fig 3B) remained infected for up to 1 week with Ymt mutant *Y*. *pestis*. The mean bacterial load of fleas infected with Ymt^−^ *Y*. *pestis* was higher for males at both 1 and 7 days after infection, but the difference was not statistically significant (Fig 3C). In contrast, infection rates were identical between sexes when infected with the wild-type parent strain (Fig 3A and 3B). Regardless of sex, the few fleas that remained infected had a lightly colonized proventriculus (≤ 25% coverage of the proventricular spines by a bacterial mass; (Fig 3D and Table 1).

### Clearance of Ymt^−^ *Y*. *pestis* correlates with the rate of RBC digestion

To confirm that the differential survival of the Ymt mutant in the flea was not due to inhibitory components in certain blood sources, *in vitro* growth of wild-type and mutant *Y*. *pestis* in defibrinated mouse blood, rat blood, or BHI-hemin broth was monitored during 24 h of incubation at 21° or 37°C. As expected, the Ymt mutant and the parental strain grew equally well in all three substrates (S2A and S2B Fig). Additionally, exposure to hemolyzed mouse RBCs or defibrinated mouse plasma did not affect bacterial viability (S2C Fig). These data indicate that the Ymt mutant phenotype observed in the flea gut is unrelated to differential growth characteristics in mouse blood and that the clearance of the Ymt mutant may require processing of the blood meal by flea digestive enzymes [4].

**Fig 4:**
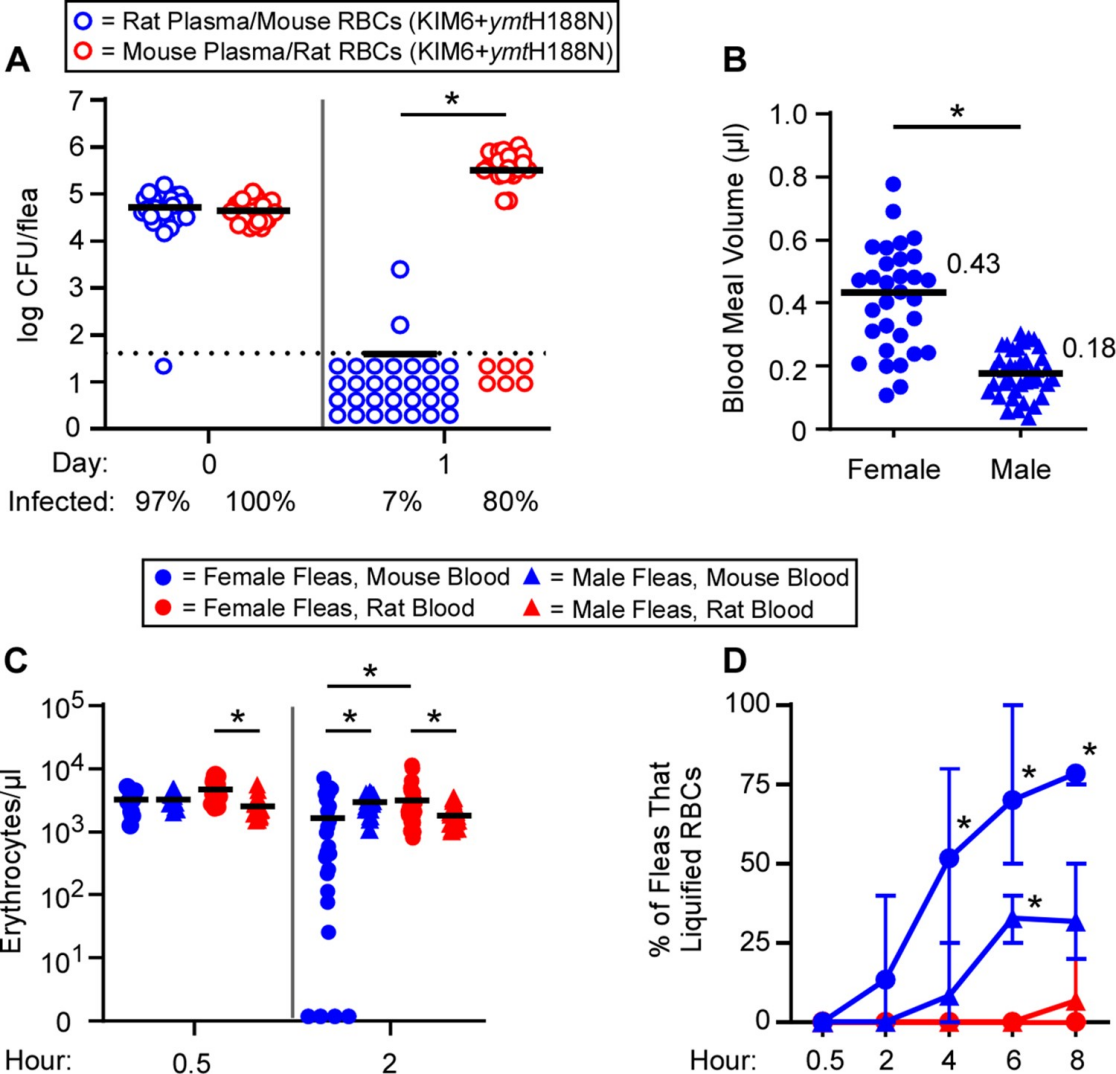
Survival of the Ymt mutant in the flea correlates with slower RBC digestion. A) Bacterial titers and infection rates for groups of female *X*. *cheopis* infected using reconstituted, plasma-swapped mouse blood (brown rat plasma mixed with mouse RBCs; blue) or brown rat blood (mouse plasma mixed with rat RBCs; red) containing 1.3x10^8^–2.8x10^8^ CFU/ml KIM6+*ymt*H188N. Data are the pooled results from 3 independent experiments (n = 10); bars represent the median. *p < 0.0001 by Mann-Whitney test. B) Blood meal volumes of individual female or male *X*. *cheopis* allowed to feed for 1 h on a neonatal mouse. Mean blood meal volumes are indicated, *p <0.0001 by Student’s t-test. C) The RBC concentration in individual *X*. *cheopis* female or male digestive tracts 0.5 or 2 h after ingestion of sterile mouse or rat blood. Bars represent the mean of 3 independent assays using n = 3–6 (0.5 h) or n = 6–10 (2 h) fleas. *p <0.05 by two-way ANOVA with Tukey’s post-test. D) The mean proportion and range of male or female *X*. *cheopis* that completely liquified sterile mouse or brown rat blood during the first 8 h of digestion. Data are from groups of 3–6 digestive tracts excised from fleas at each timepoint and condition from 3 independent experiments; n = 9–15. *p <0.05 by Fisher’s exact test compared to rat blood group. A representative image series of these data is shown in S3 Fig.

To determine the fraction of blood responsible for clearance of the Ymt mutant in the flea gut, we infected *X*. *cheopis* using reconstituted, plasma-swapped mouse or brown rat blood (rat plasma mixed with mouse RBCs or vice versa) containing KIM6+*ymt*H188N *Y*. *pestis*. One day after infection, only 7% of fleas infected using brown rat plasma with mouse RBCs remained infected compared to 80% of those infected using mouse plasma and brown rat RBCs (Fig 4A). In sum, addition of brown rat plasma to mouse RBCs did not rescue the Ymt mutant in the flea gut, and addition of mouse plasma to rat RBCs did not result in impaired bacterial infectivity. These results indicate that the bactericidal agent is primarily produced as a consequence of digestion of RBCs, such as those from a mouse, and that the contribution of plasma to the Ymt^−^ strain phenotype is likely modest or inconsequential.

Given that Ymt appeared to protect against a bactericidal product of RBC digestion, we decided to test whether female and male *X*. *cheopis* digested mouse and rat RBCs at equivalent rates. First, to get a better understanding of flea digestion kinetics between flea sexes, we determined that female fleas ingest, on average, roughly twice as much blood as male fleas (Fig 4B). Next, we found that the RBC concentration in the flea gut was similar, regardless of blood source or flea sex, within the first 30 minutes after the bloodmeal (Fig 4C). However, by two hours after feeding, female fleas that ingested mouse blood had the largest reduction in red cell counts. The majority of mouse RBCs had lysed by 2 h in ~1/3^rd^ of female fleas, whereas all other flea sex-blood source combinations showed a lower RBC digestion rate (Fig 4C). Beyond 2 h, both mouse and rat RBCs frequently aggregated in large clusters in the flea digestive tract, rendering hemocytometer counts unfeasible. To address this, we imaged digestive tracts excised from fleas every 2 h for the first 8 h after an uninfected blood meal. We found that most female fleas completely digest and liquify mouse RBCs within 4–6 h (Figs 4D and S3). At 6 h, the gut of 70% of the female fleas (9 of 13) contained only a moderately viscous pink fluid, devoid of cellular material. Male fleas took longer to digest mouse blood; after 6–8 h of digestion, only ~30% had completely liquified their blood meal (Figs 4D and S3). In contrast, brown rat blood took considerably longer for both male and female fleas to digest. By 6 h after feeding, the digestive tract always contained a thick, viscid, brownish-red slurry of aggregated RBC stroma in various stages of breakdown, distinctly more viscous than what was present in fleas fed mouse blood (S3 Fig). By 8 h, no female fleas (0 of 11) and only 7% of males (1 of 15) had completely liquified their brown rat blood meal (Fig 4D). The relative amount of solid material in the fleas fed brown rat blood remained fairly constant over the first 8 h of digestion, indicating that both male and female fleas typically require more than 8 h to liquify brown rat blood.

The identical temporal patterns of RBC digestion were also observed in fleas infected with Ymt^−^ *Y*. *pestis*. By 24 h after infection, fleas infected using brown rat blood routinely contained significant quantities of undigested midgut material (Fig 3D). In contrast, fleas infected using mouse blood typically contained only the viscous pink or red liquid, with little to no solid material (Fig 3D). Collectively, these data indicate a correlation between the rate of RBC digestion and the clearance of Ymt^−^ *Y*. *pestis* from the flea gut.

### Ymt^−^ *Y*. *pestis* can be transmitted beyond the early phase when fleas are infected using brown rat blood

**Fig 5:**
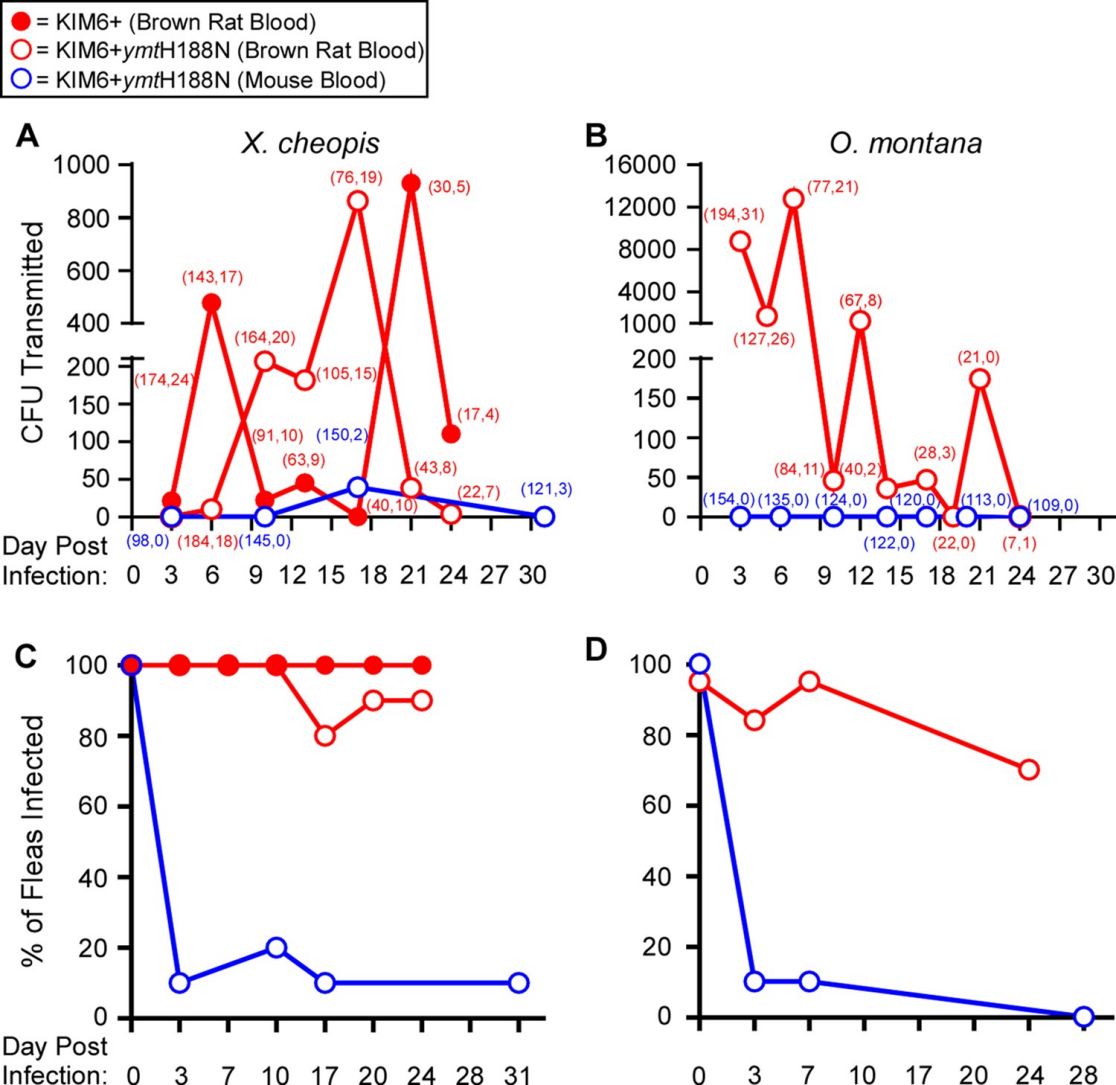
Rodent fleas can transmit Ymt mutant *Y*. *pestis* for at least 3 weeks when infected using brown rat blood. *Y*. *pestis* transmission dynamics were monitored for 3 to 4 weeks for groups of 150–267 *X*. *cheopis* (A) or *O*. *montana* fleas infected using 3.4 x 10^8^−1.9 x10^9^ CFU/ml KIM6+ or KIM6+*ymt*H188N *Y*. *pestis* (B) in either mouse (blue) or brown rat (red) blood and subsequently maintained on sterile blood of the same type. Numbers in parentheses indicate the total number of fleas that fed followed by the number of fleas with evidence of foregut obstruction (partially or fully blocked). Roughly equivalent numbers of male and female fleas were used for transmission assays. Infection rate was determined for groups of 10–20 female C) *X*. *cheopis* or D) *O*. *montana* at various times following infection.

To assess transmission of the mutant strain, groups of *O*. *montana* or *X*. *cheopis* fleas were infected with the Ymt mutant or the parent strain in either brown rat or mouse blood and were fed periodically on sterile blood of the same source. After each maintenance feed, the blood was collected from the feeding device and plated to determine the number of CFUs transmitted. Early-phase transmission (3 days post-infection) of Ymt mutant *Y*. *pestis* was detected for *O*. *montana* infected using brown rat blood, but not if mouse blood was used (Fig 5A and 5B). Furthermore, *X*. *cheopis* and *O*. *montana* infected using brown rat blood transmitted moderate to high levels of the Ymt mutant for at least 2 or 3 weeks, respectively, during the biofilm-dependent phase of transmission. Transmission of the Ymt mutant and the parental Ymt^+^ *Y*. *pestis* strains by *X*. *cheopis* infected using brown rat blood was roughly comparable (Fig 5A). In contrast, fleas infected using mouse blood rarely became blocked and only a single instance of transmission was observed (*X*. *cheopis*, day 17), in which very few CFU were transmitted (Fig 5A). Infection rates of fleas used for transmission tests were similar to those shown in Figs 2 and S1 (Fig 5C and 5D). Reduced transmission by fleas infected using brown rat blood during the later weeks of infection may be partially attributable to the higher mortality rate of these fleas. Greater than 80% of both flea species had died by 3 weeks after infection. The elevated mortality may be attributable to the more severe *Y*. *pestis* infection in the foregut of fleas infected using brown rat blood (Figs 1 and 5) [14]. The overall incidence of foregut obstruction (fully and partially blocked fleas) was 39–45% for rodent fleas infected and maintained on brown rat blood but only 0–2.5% for those infected using mouse blood (Fig 5). Consistent with our finding that male *X*. *cheopis* are more susceptible to infection by Ymt^**−**^ *Y*. *pestis* in mouse blood (Fig 3), all 5 fleas in this group that became blocked were males (Fig 5A).

## Discussion

Gene gain and gene loss were both major drivers of the recent evolutionary emergence of *Y*. *pestis*, and acquisition of Ymt was critical for the transition to a flea-borne life cycle because it greatly enhanced survival of *Y*. *pestis* in the flea gut [15]. The original report of the protective effect of Ymt hypothesized that the Phospholipase D activity of Ymt directly or indirectly protects *Y*. *pestis* against a bactericidal byproduct of blood digestion [4], and was based mainly on female fleas infected using mouse blood. We extend that original characterization here, showing that the protective function of Ymt is much less important when fleas feed on bacteremic brown rat blood than on bacteremic mouse, human, or black rat blood. With brown rat blood, flea infection and proventricular blockage rates were not significantly different for the first few days of infection and only slightly reduced after 1 week for Ymt^−^ compared to Ymt^+^ *Y*. *pestis*, whereas these rates were greatly reduced in the first 24 h of infection for the Ymt mutant using the other blood sources. Correspondingly, both *X*. *cheopis* and *O*. *montana* fleas infected using brown rat blood transmitted Ymt^**−**^ *Y*. *pestis* relatively efficiently, whereas as predicted by the previous study [4] fleas infected using mouse blood rarely transmitted, and few CFU were transmitted.

Other major findings of this study are that Ymt likely protects *Y*. *pestis* from a product of RBC digestion, and not a plasma digestion product as hypothesized previously [4]; and that the importance of Ymt correlates with RBC digestion kinetics. Previous studies of RBC digestion by female *X*. *cheopis* demonstrated that fleas digest their blood meals more rapidly than many other blood-feeding arthropods, such as mosquitos and ticks, and that the digestive tract expresses a number of trypsin-like transcripts within the first hours following feeding [16,17]. Electron microscopic analysis of the *X*. *cheopis* midgut epithelium indicates that secretory vesicles, likely containing digestive enzymes, are produced in advance of feeding and are released more or less immediately following ingestion of blood [18]. However, we found that the digestion rate can vary depending on the host blood source. Brown rat RBCs were digested more slowly and incompletely than mouse RBCs. Fleas infected using brown rat blood routinely had large quantities of undigested material in their gut 24 h after infection, whereas fleas infected using mouse blood were essentially devoid or had greatly reduced amounts of solid blood material. In addition, our results show that female *X*. *cheopis* digest mouse blood more rapidly than males do, despite ingesting roughly twice as much blood. Digestion kinetics often differ between insects of the opposite sex, as oviposition and egg maturation are physiologically associated with digestion, and females typically have greater energetic demands due to these biological imperatives [19,20]. The digestion patterns we observed for female fleas and mouse blood, in which most erythrocytes are digested during the first few hours, are consistent with previous estimates [16]. Other microscopic analyses of flea gut contents also have indicated that *X*. *cheopis* females digest mouse blood more rapidly than males [21]. Overall, the survivability of Ymt^−^ *Y*. *pestis* after being ingested by a flea correlated well with RBC digestion rate: good, nearly normal survival in both sexes with rat blood infections; and intermediate survival in male fleas but poor survival in female fleas with mouse blood infections.

In addition to digestive enzymes, RNA-Seq analysis of the *X*. *cheopis* digestive tract transcriptome revealed that antimicrobial peptides are rapidly produced in response to ingestion of *Y*. *pestis* [17]. However, Ymt does not appear to have a role in protection against the flea immune response. *Drosophila*, an insect that has been developed as a surrogate *Y*. *pestis* infection model, produces a diverse array of antimicrobials in response to Gram-negative bacteria, yet Ymt mutant strains show no defect in fruit fly colonization or bacterial burden [22]. In this model, *Drosophila* larvae ingest *Y*. *pestis*-laden cornmeal agar, rather than blood, to initiate infection [23], suggesting that the Ymt mutant colonization defect in the flea is uniquely related to blood digestion rather than to insect innate immunity. Furthermore, the Ymt mutant shows no enhanced susceptibility to common antimicrobials that target the outer membrane (polymyxin B, SDS, lysozyme, etc.) or to other potentially bacteriolytic enzymes and environmental stressors that would be encountered in the arthropod gut environment (proteases, lipases, osmotic and oxidative stress) [24]. It was previously hypothesized that the Phospholipase D activity of Ymt provides protection against a bacteriolytic byproduct of blood digestion by either modifying the bacterial envelope to make the bacteria resistant to lysis (prophylaxis model), or by direct or indirect neutralization of the lytic agent (antidote model) [24]. While we have not resolved the mechanism by which Ymt provides protection to the bacteria, our data further indicate that it is a byproduct of RBC digestion that induces the abnormal spheroplast morphology indicative of cell envelope damage to the Ymt^−^ mutant in the flea. Notably, Ymt^−^ *Y*. *pestis* grows normally in all blood sources during *in vitro* growth assays, and can produce septicemic plague in mice [8].

Based on these findings, we propose a nuanced model for the role of Ymt in flea-borne transmission. When fleas are infected from a host (*e*.*g*. mouse) whose RBCs are digested rapidly, the bactericidal byproduct generated reaches cytotoxic levels within the first few hours and eradicates Ymt^−^ *Y*. *pestis* from the midgut. This is more pronounced in female fleas, which take larger blood meals and digest them more rapidly than males. A significantly higher percentage of male *X*. *cheopis* become infected after feeding on mouse blood containing Ymt^**−**^ *Y*. *pestis*, and the infection can involve the midgut and the proventriculus. In females, only those few fleas in which the mutant localizes to the foregut, sequestered from the digestive milieu of the midgut, remain colonized [4]. In contrast, when fleas are infected from a host (*e*.*g*. brown rat) whose RBCs are digested slowly, we hypothesize that the bactericidal byproduct does not reach lethal levels before the bacteria have time to coalesce into large dense aggregates. These aggregates develop in the midgut and proventriculus within a few hours after ingestion and appear to be surrounded by a viscous matrix [25,26], suggesting that they may be protected from exposure to bactericidal factors in the midgut. Supporting this idea is the observation that providing fleas infected using brown rat blood with two maintenance mouse blood meals 2–7 days later did not significantly reduce their high infection rates, which remained comparable to those seen for fleas provided brown rat blood maintenance meals. This model could also account for the disparity in infection rates between male and female fleas infected with Ymt^**−**^ *Y*. *pestis* using mouse blood. Our results suggest that the bactericidal agent is produced in the flea gut regardless of the host blood ingested, but the digestion kinetics of the various blood sources dictate the frequency and rate at which an absolute lethal concentration is achieved relative to the time it takes for *Y*. *pestis* to coalesce into large dense masses. Although the model emphasizes RBC digestion kinetics, it is also possible that biochemical differences between mouse and rat RBCs contribute to the much greater sensitivity of the Ymt^−^ mutant to mouse RBC digestion.

**Fig 6:**
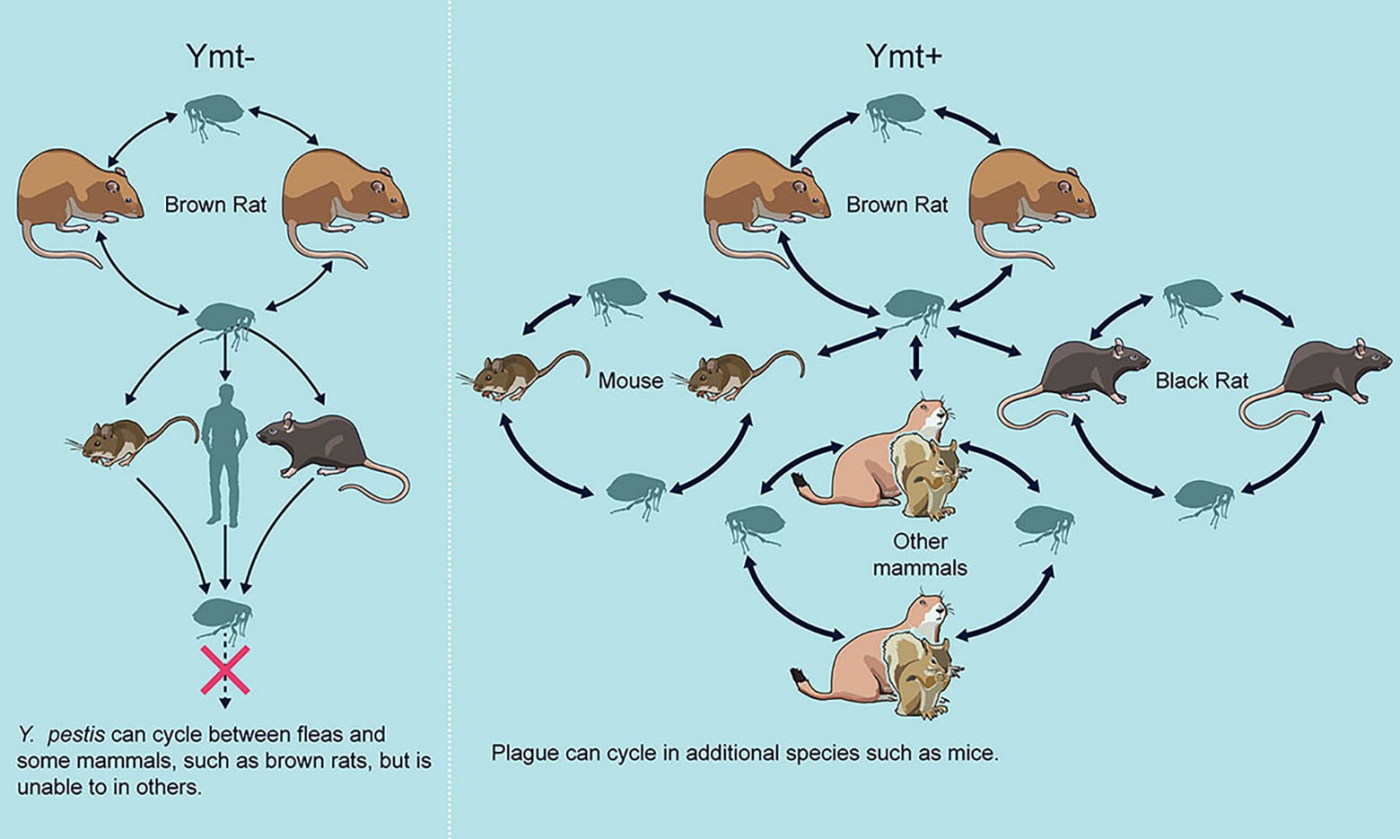
Model of the adaptive function of Yersinia murine toxin (Ymt) during the evolution of the flea-borne life cycle of *Y*. *pestis*. In this model, ancestral *Y*. *pestis* strains lacking the *ymt* gene (Ymt^−^; left) could cycle between fleas and certain species of rodent with flea-colonization-permissive host blood, such as brown rats (*Rattus norvegicus*), but not those with non-permissive blood, such as mice (*Mus* spp). Following acquisition of *ymt* on the pMT1 plasmid (Ymt+ strains; right), the progenitor of modern, extant strains of *Y*. *pestis* was able to stably colonize fleas that fed on bacteremic hosts with a blood chemistry that is not permissive for Ymt-negative strains. Thus, acquisition of *ymt* effectively greatly expanded the range of mammalian hosts that could support a flea-mammal transmission cycle. Although male fleas become infected at a moderate rate with Ymt^−^ *Y*. *pestis* infected with non-permissive mouse blood (Fig 3), their potential to transmit is likely not sufficient to maintain a stable transmission cycle (Fig 5).

These results suggest a revision to the evolutionary history of *Y*. *pestis* (Fig 6). Ancestral strains that had not yet acquired *ymt*, such as those circulating in the Neolithic and Bronze Age, have been thought to be fully virulent for mammals but incompetent for flea-borne transmission [27–30]. However, our data indicate that they could have been maintained in stable flea-borne transmission cycles among brown rats and other hosts with similarly permissive blood characteristics. In addition to early-phase transmission, which does not require Ymt following rat blood infection [13], transmission by the later, proventricular blockage mechanism would be robust within these host populations. Acquisition of *ymt*, however, would have been adaptive for two reasons. First, it modestly augments flea infectivity even when the bacteremic host blood has a largely permissive biochemical profile, such as the blood of the brown rat. More consequentially, Ymt enzymatic activity greatly enhances the percentage of fleas that develop a chronic, transmissible infection when *Y*. *pestis* is acquired from a host with blood biochemistry that was (originally) poorly permissive for flea infection (e.g., mice, humans, black rats) (Fig 6). The ancestral *Y*. *pestis* lineages that lacked *ymt* are extinct, suggesting that host restriction and reduced flea transmissibility of these strains contributed to reduced Darwinian fitness and their eventual disappearance [31]. It’s tempting to hypothesize that rodents involved in ancestral plague transmission cycles have blood biochemistry similar to that of the brown rat. For example, Tarbagan marmots (*Marmota siberica*) have been proposed as host to the original *Y*. *pestis* clone, if so, their blood would likely support chronic flea infection by Ymt^−^ *Y*. *pestis* strains [32].

Collectively, our results indicate that acquisition of Ymt did not allow *Y*. *pestis* to colonize fleas *per se*, but significantly improved *Y*. *pestis* survival in the flea gut in the context of RBC digestion and processing kinetics of blood meals from different mammals. In effect, acquisition of Ymt greatly expanded the range of hosts that could support a stable mammal-flea transmission cycle (Fig 6). The antibacterial product of RBC digestion and the mechanism of Ymt-mediated resistance to it remain to be determined. However, this study provides an important update and revision to Ymt’s adaptive function during the recent evolutionary transition of *Y*. *pestis* to a flea-borne pathogen involving the ecologically broad range of mammals that characterizes modern strains.

## Methods

### Ethics statement

Experiments involving animals were approved by the Rocky Mountain Laboratories, National Institute of Allergy and Infectious Diseases, National Institutes of Health Animal Care and Use Committee (Animal Protocol #2019–011E) and were conducted in accordance with all National Institutes of Health guidelines.

### Bacterial strains and plasmids

*Y*. *pestis* strains, plasmids, and primers used in this study are listed in Table 2. *Y*. *pestis* KIM6+ *ymt*H188N expresses a non-functional Ymt with a point mutation in one of the two HKD catalytic domains that typify this class of phospholipase D enzymes [33] and is referred to here as the Ymt^−^ or Ymt mutant strain. pCH16 (referred to hereafter as pYmt) contains the wild type *ymt* gene expressed by its native promoter and was used for complementation of the *ymt*H188N mutant [8]. All *Y*. *pestis* strains were transformed with pAcGFP1 (Clontech/Takara Bio) or pGFP-Km^r^ (this study), respectively. pGFP-Km^r^ was used for strains complemented with pCH16 to maintain selection for both plasmids prior to use in flea infections.

**Table 2:** Strain and Plasmid List.

Strain/Plasmid	Key Properties	Reference
***Y*. *pestis* strains**		
KIM6+	pCD1^-^, pMT1+, pPCP1+, Pgm+	[34,35]
KIM6+*ymt*H188N	KIM6+ modified to express a non-functional Ymt with a point mutation in one of the two HKD catalytic domains.	[33]
**Plasmids**		
pAcGFP1	Ap^r^, constitutively expresses GFP	Clontech/Takara Bio (Mountain View, CA)
pGFP-Km^r^	pAcGFP1 was amplified by inverse PCR to selectively exclude the *bla* gene and replace it with a SacI site. A kanamycin resistance cassette with terminal SacI sites was inserted into the linear inverse PCR product of pAcGFP1, which was then religated.	This Study
pCH16 (pYmt)	Ap^r^, expresses *ymt* from its native promoter.	[8]
**Primers**		
pGFP-Km^r^ (Inverse PCR)	F: CGTCGAGCTCTTCGTTCCACTGAGCGTCA R: CGTAGAGCTCGTACAATCTGCTCTGATGCCG	This Study
pGFP-Km^r^ (Km^r^ Casette)	F: CGTAGAGCTCTCCAGCCAGAAAGTGAGGGAG R: GCATGAGCTCGGGAAAGCCACGTTGTGTCTC (Amplified from pKD4; [36])	This Study

pCD1 virulence plasmid, encodes type 3 secretion system

pMT1 plasmid, encodes the phospholipase D Yersinia murine toxin (Ymt) and capsule antigen (F1)

pPCP1 plasmid, encodes plasminogen activator/protease (Pla), the bacteriocin pesticin (Pst), and pesticin immunity protein (Pim)

Pgm pigmentation locus and pathogenicity island, encodes the hemin storage locus (*hmsHFRS* operon) and iron acquisition genes

Ap^r^ ampicillin resistance; Km^r^ kanamycin resistance

### Flea infection and host blood

Prior to infection, *X*. *cheopis* or *O*. *montana* fleas were randomly pulled from colonies established at the Rocky Mountain Laboratories and starved for three days. *Y*. *pestis* strains were grown in brain-heart infusion (BHI) broth with appropriate antibiotic selection as described previously [37]. Briefly, 100 ml *Y*. *pestis* cultures, grown at 37°C overnight, were centrifuged and the bacterial pellet resuspended in 1 ml sterile PBS. Bacterial suspensions were added to a final concentration of ~5x10^8^ CFU/ml to 5 ml of heparinized Swiss-Webster mouse blood, defibrinated Sprague-Dawley brown rat (*R*. *norvegicus*) or human blood (both from BioIVT, New York), or to heparinized wild black rat (*R*. *rattus*) blood collected and shipped overnight by Alameda County, CA, Vector Control personnel. Prior to use in flea infections, black rat blood was treated with carbenicillin (100 μg/ml) and plated on 5% sheep blood agar to ensure sterility. The blood and bacterial mixture was added to a membrane feeding apparatus and groups of fleas were allowed to feed for 1 h [4]. Fleas (approximately equal numbers of males and females) that took an infectious blood meal were collected and kept at 21°C in a humidified chamber (75% RH). These fleas were provided maintenance feedings on neonatal mice 2 to 3 days after infection and again 6 to 7 days after infection. Following each maintenance feed, fleas were screened for the presence of fresh red blood in the esophagus, a condition of fleas with partial or complete blockage or PIER. At 0, 1, and 7 days following infection, 10 to 20 infected fleas were frozen at -80°C for later determination of infection status and bacterial load per flea by plating individual triturated fleas in BHI soft agar overlays as previously described [38].

For flea infections using plasma-swapped blood, the plasma fraction was separated from mouse or rat red blood cells (RBC) following centrifugation at 3000 rpm, the RBCs were washed 3 times with an equivalent volume of sterile PBS, and whole blood was reconstituted with heterologous plasma from the other rodent.

### Dissection and imaging of flea digestive tracts

Fleas infected with KIM6+*ymt*H188N suspended in mouse or brown rat blood were dissected one day after infection to determine the localization of bacteria in the digestive tract and their phenotype. The severity of proventricular infection was scored as light, moderate, or heavy as described previously [38]. Images of flea digestive tracts and bacterial biofilms were taken with a Nikon Eclipse E800 microscope equipped with a DP72 Olympus camera (Center Valley, PA) and a G-2E/C (540/25 EX) fluorescent filter (Nikon), and were processed using Olympus cellSens software.

### Blood meal volume and red blood cell digestion rate

For blood meal volume determination, individual adult *X*. *cheopis* not fed for 5 days prior, were weighed using a Sartorius SC 2 Microbalance (Goettingen, Germany) before and immediately after feeding on a neonatal mouse. Fleas were anesthetized with CO_2_ and placed in a microcentrifuge tube prior to each weighing. Bloodmeal weight was determined by subtracting the pre-feed weight from the post-feed weight and then converted to volume based on the specific gravity of mouse blood [39].

To assess digestion kinetics, *X*. *cheopis* were allowed to feed on sterile Swiss Webster mouse or Sprague-Dawley rat blood for 30 minutes. Digestive tracts were dissected from groups of male or female fleas immediately (0.5 h) following feeding or 2 h after feeding. Excised digestive tracts were placed in 20 μl sterile PBS on a microscope slide and expressed with forceps to release midgut contents. Expelled gut contents were diluted 1:5 in PBS containing 0.4% (w/v) trypan blue, mixed, and the number of RBCs determined using a hemocytometer.

For the digestion image series, fleas were fed as described above and digestive tracts were imaged using a Nikon SMZ1500 dissection microscope with a DP72 Olympus camera. Digestive tracts were visually scored for the presence or absence of cellular material to determine if they had completely liquified the blood meal.

### Transmission assays

Transmission by *O*. *montana* or *X*. *cheopis* fleas was assayed as described previously with minor modifications [40]. Fleas infected as described above were refed using the artificial feeding system every 3 days following infection on sterile host blood of the same source they were initially infected with. After each feed (except for the *X*. *cheopis*-mouse blood experiment in which transmission was assessed every 6 days following the initial assay on day 3) the entirety of the blood from the feeding reservoir was collected and distributively spread onto blood agar-carbenicillin plates. In addition, the feeding reservoir was washed 6 times with 5 ml of sterile PBS; these washes were combined, centrifuged, and the resulting pellet resuspended in 2 to 3 ml of PBS and plated. Blood agar plates were incubated for 48 h at 28°C and GFP positive colonies counted to determine the number of CFUs transmitted.

### Bacterial in vitro growth and susceptibility assays

*Y*. *pestis* strains were grown in BHI containing 10 μg/ml hemin. After 18 h incubation at 28°C without shaking, cultures were diluted to an OD600 of 0.1, centrifuged at 6000 rpm for 10 min, and the bacterial pellets resuspended in an equal volume of sterile PBS. *Y*. *pestis* was then added to 10 ml of BHI-hemin, defibrinated brown rat blood, or defibrinated mouse blood to a final concentration of ~1x10^6^ CFU/ml and cultures were incubated in 50 ml conical tubes at either 21° or 37°C without shaking. After 0, 2, 4, 6, 8, and 24 h incubation the cultures were mixed well and a 100 μl sample was removed, serially diluted, and plated on blood agar for CFU determination.

For susceptibility assays, ~1x10^6^ *Y*. *pestis*, prepared as above, were added to 1 ml of defibrinated mouse plasma, a suspension of lysed mouse RBCs, or BHI broth in an 8-well culture dish. Lysed mouse RBCs were prepared by mixing washed cells 1:1 with sterile PBS followed by three freeze-thaw cycles. After 1 h at 25°C, 10-fold serial dilutions of the suspensions were plated on blood agar. The percentage of CFU recovered from each medium relative to the BHI control was calculated to assess antibacterial activity.

## Supporting information

S1 Fig The *Y*. *pestis* Ymt mutant phenotype in female *Oropsylla montana* fleas is identical to that in *X*. *cheopis*. Groups of *O*. *montana* fleas that fed on mouse or brown rat blood containing 2.8 x 10^8^–7.1 x 10^8^ CFU/ml *Y*. *pestis* KIM6+, KIM6+*ymt*H188N, or KIM6+*ymt*H188N(pYmt) were screened for 1 week for A) the percentage of fleas that remained infected; B) development of a foregut obstruction that interfered with normal blood-feeding; and C) bacterial burden. Data are the results from 3 (KIM6+*ymt*H188N groups) or 1–2 (KIM6+ and KIM6+*ymt*H188N(pYmt) groups) independent experiments. Samples consisted of 9–20 female fleas (A, C) or 40 to 112 fleas (approximately equal numbers of males and females; B) per experiment. The mean and standard error (A, B) or median (C) are indicated. *p <0.05 by chi-square (A, B) or by Kruskal-Wallis test with Dunn’s post-test (C).
(TIF)

S2 Fig The Ymt mutant has no growth or survival defects under *in vitro* conditions. Growth kinetics of *Y*. *pestis* KIM6+ and KIM6+*ymt*H188N grown in mouse blood, brown rat blood, or BHI broth supplemented with hemin and incubated for 24 h at A) 21°C or B) 37°C. The mean and standard error of 3 independent experiments are shown. C) Bacterial survival assay in which 1x10^6^ CFU KIM6+*ymt*H188N were added to BHI broth, defibrinated mouse plasma, lysed mouse red blood cells and incubated for 1 h at 25°C. Dilutions of each medium were then plated to determine CFU concentrations. The mean and standard error of 3 independent experiments are shown and expressed as the percent CFU recovered relative to the BHI control.
(TIF)

S3 Fig *X*. *cheopis* blood source- and sex-related differences in red blood cell digestion rates. Representative image series of the data shown in Fig 4D. Digestive tract preparations were scored for the presence or absence of particulates that exuded from the flea midgut into the surrounding saline. Mouse blood meals were completely liquified by most female fleas in 4–6 h (far left) but partially digested RBC stroma were still present in most males for 6–8 h (middle left). With rare exception, fleas that ingested sterile rat blood, regardless of sex, contained a fairly stable amount of partially digested RBCs for at least 8 h following feeding (right). Scale bar = 100 μm.
(TIF)

S1 Data File containing numerical data used for Figs 1–5, S1 and S2. (XLSX)
