# Supplementary figures and images for: Acquisition of yersinia murine toxin enabled Yersinia pestis to expand the range of mammalian hosts that sustain flea-borne plague
Source: PLoS Pathog. 2021 Oct 14;17(10):e1009995. doi: 10.1371/journal.ppat.1009995 (PMC8547695; doi:10.1371/journal.ppat.1009995)

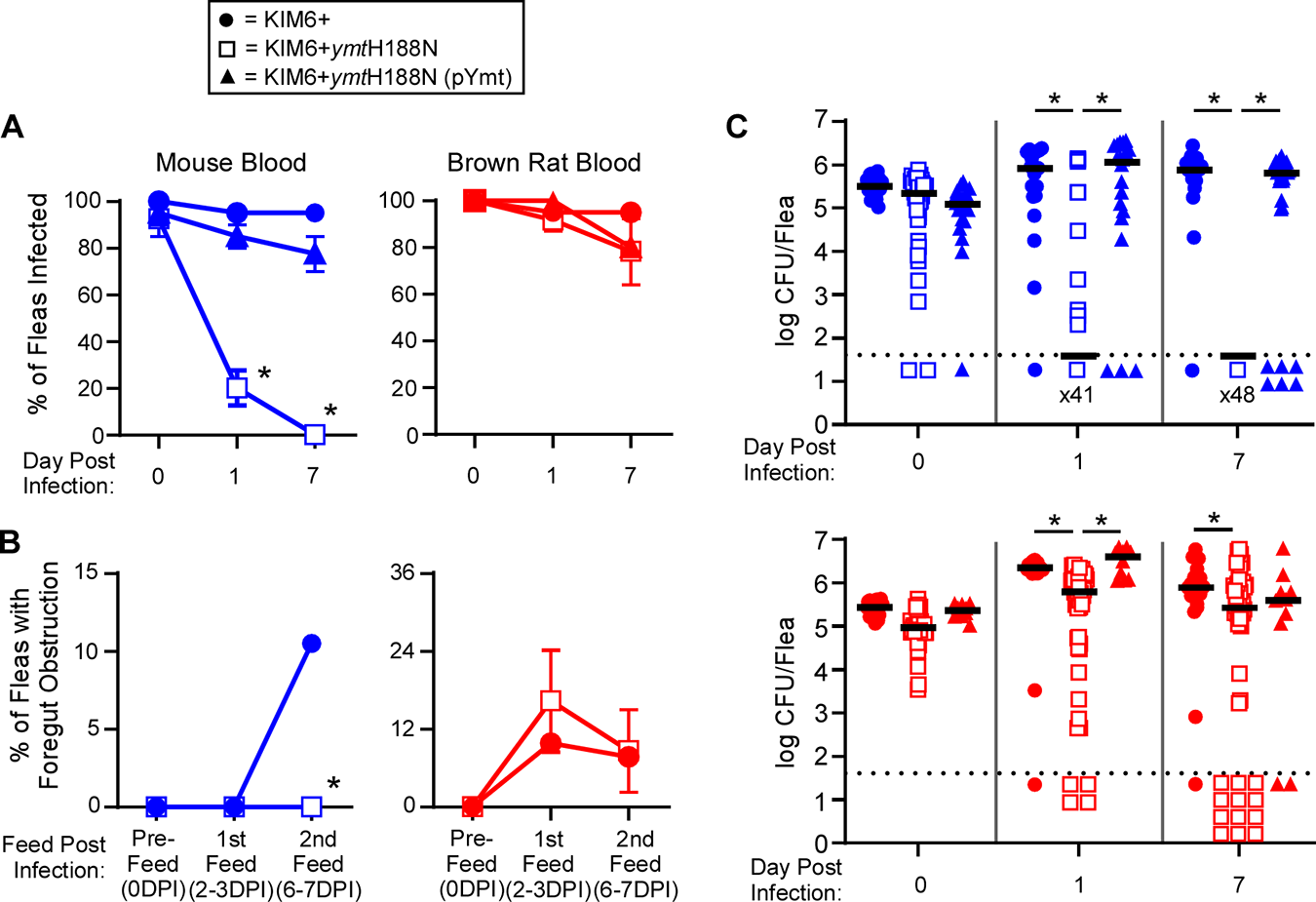

Supplement: S1 Fig — Groups of O. montana fleas that fed on mouse or brown rat blood containing 2.8 x 10^8^–7.1 x 10^8^ CFU/ml Y. pestis KIM6+, KIM6+ymtH188N, or KIM6+ymtH188N(pYmt) were screened for 1 week for A) the percentage of fleas that remained infected; B) development of a foregut obstruction that interfered with normal blood-feeding; and C) bacterial burden. Data are the results from 3 (KIM6+ymtH188N groups) or 1–2 (KIM6+ and KIM6+ymtH188N(pYmt) groups) independent experiments. Samples consisted of 9–20 female fleas (A, C) or 40 to 112 fleas (approximately equal numbers of males and females; B) per experiment. The mean and standard error (A, B) or median (C) are indicated. *p <0.05 by chi-square (A, B) or by Kruskal-Wallis test with Dunn’s post-test (C). (TIF) [file ppat.1009995.s001.tif]

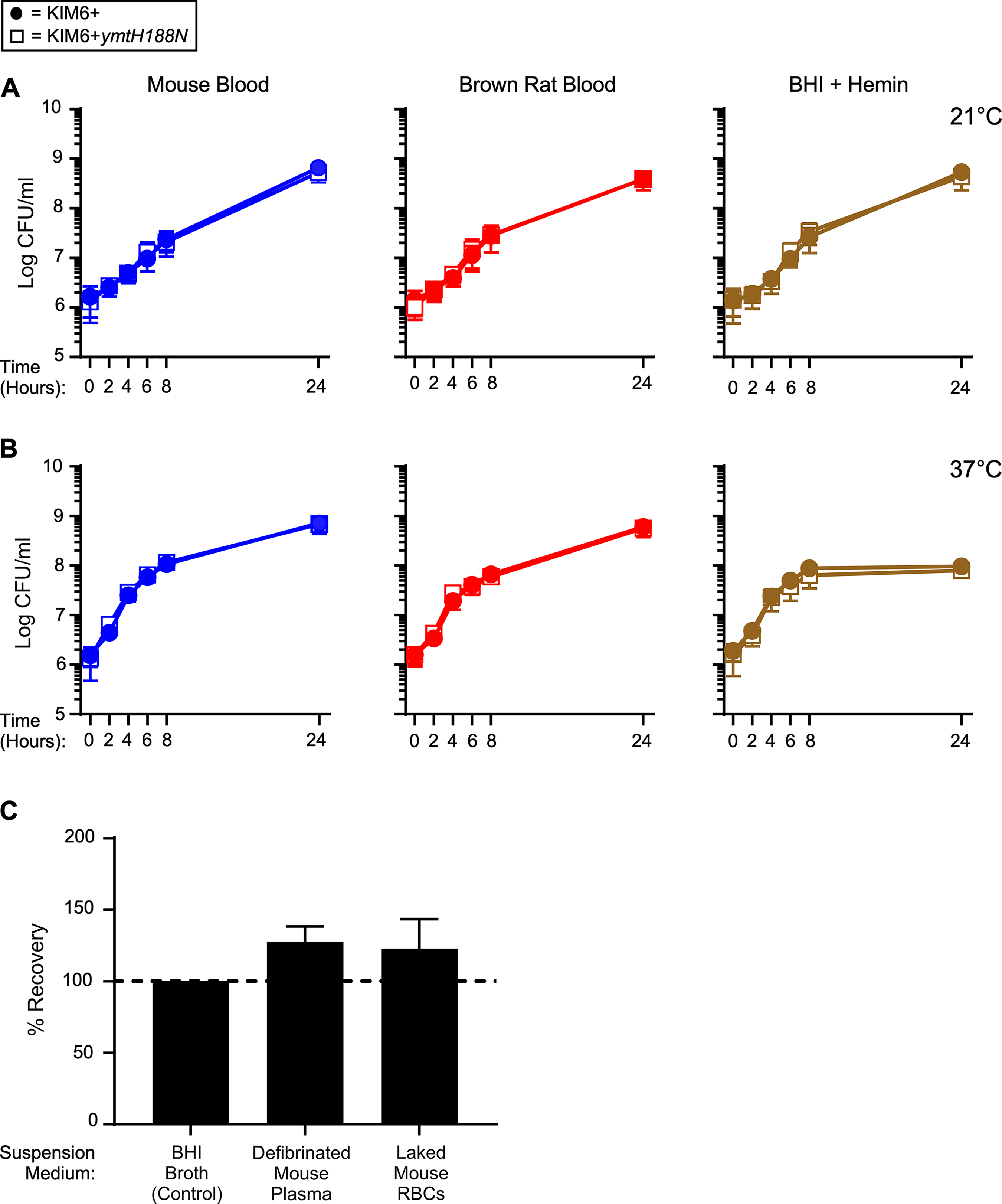

Supplement: S2 Fig — Growth kinetics of Y. pestis KIM6+ and KIM6+ymtH188N grown in mouse blood, brown rat blood, or BHI broth supplemented with hemin and incubated for 24 h at A) 21°C or B) 37°C. The mean and standard error of 3 independent experiments are shown. C) Bacterial survival assay in which 1x10^6^ CFU KIM6+ymtH188N were added to BHI broth, defibrinated mouse plasma, lysed mouse red blood cells and incubated for 1 h at 25°C. Dilutions of each medium were then plated to determine CFU concentrations. The mean and standard error of 3 independent experiments are shown and expressed as the percent CFU recovered relative to the BHI control. (TIF) [file ppat.1009995.s002.tif]

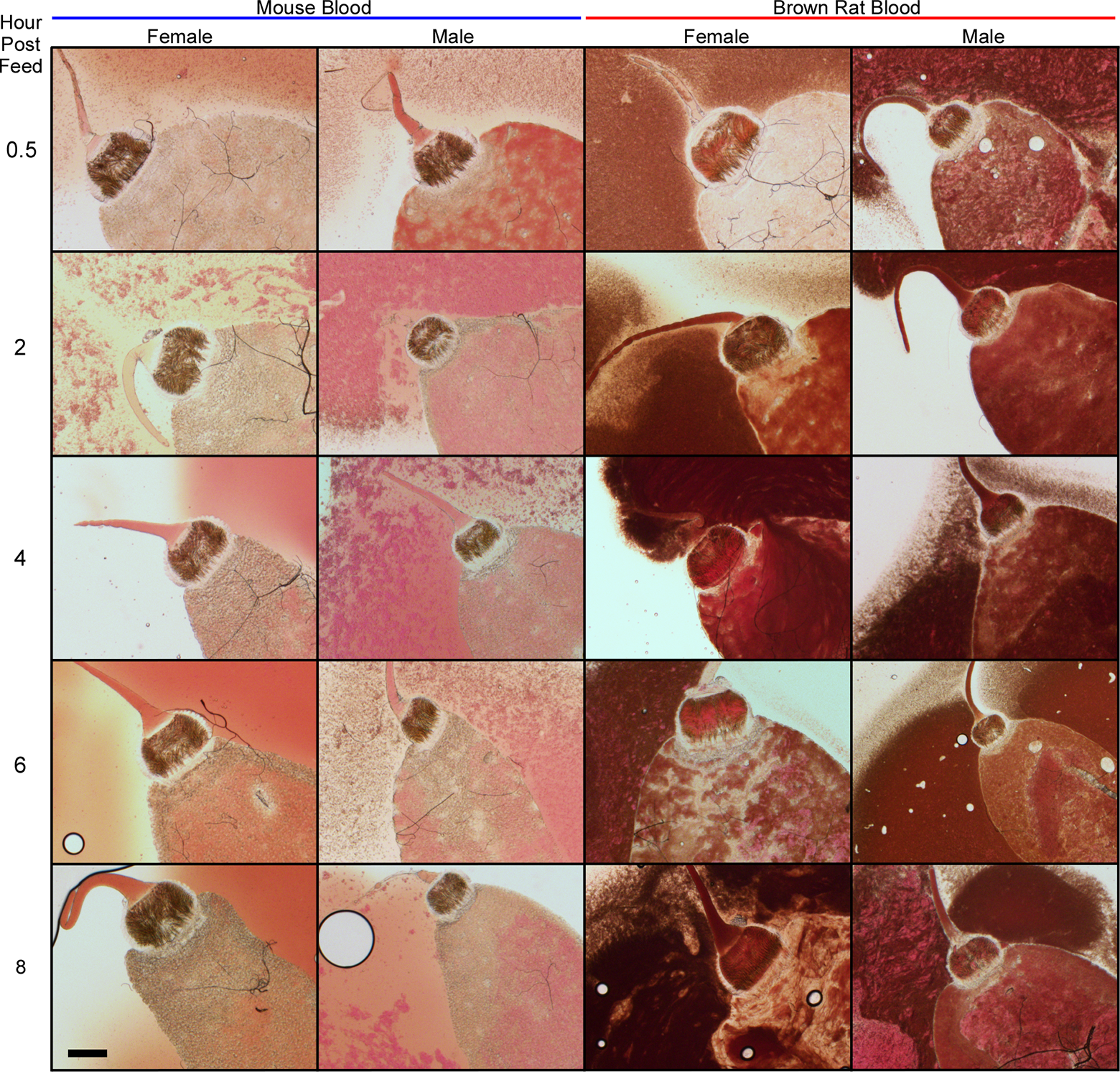

Supplement: S3 Fig — Representative image series of the data shown in Fig 4D. Digestive tract preparations were scored for the presence or absence of particulates that exuded from the flea midgut into the surrounding saline. Mouse blood meals were completely liquified by most female fleas in 4–6 h (far left) but partially digested RBC stroma were still present in most males for 6–8 h (middle left). With rare exception, fleas that ingested sterile rat blood, regardless of sex, contained a fairly stable amount of partially digested RBCs for at least 8 h following feeding (right). Scale bar = 100 μm. (TIF) [file ppat.1009995.s003.tif]
